# Exploring factors associated with ART adherence and retention in care under Option B+ strategy in Malawi: A qualitative study

**DOI:** 10.1371/journal.pone.0179838

**Published:** 2017-06-21

**Authors:** Salem Gugsa, Katy Potter, Hannock Tweya, Sam Phiri, Odala Sande, Pascal Sikwese, Janet Chikonda, Gabrielle O’Malley

**Affiliations:** 1 Lighthouse Trust, Lilongwe, Malawi; 2 International Training and Education Center for Health (I-TECH), University of Washington Department of Global Health, Seattle, Washington, United States of America; 3 The International Union Against Tuberculosis and Lung Disease, Paris, France; 4 University of North Carolina, School of Medicine, Department of Medicine, Chapel Hill, North Carolina, United States of America; 5 University of Malawi, College of Medicine, School of Public Health and Family Medicine, Department of Public Health, Lilongwe, Malawi; 6 Ministry of Health, District Health Office, Lilongwe, Malawi; San Antonio Military Medical Center, UNITED STATES

## Abstract

Although several studies have documented challenges related to inadequate adherence to antiretroviral therapy (ART) and high loss to follow-up (LTFU) among Option B+ women, there is limited understanding of why these challenges occur and how to address them. This qualitative study examines women’s experiences with ART adherence and retention in care. Between July and October 2015, in-depth interviews were conducted with 39 pregnant and lactating women who initiated ART at Bwaila Hospital in Lilongwe, Malawi. Study participants included 14 in care and 25 out of care women, according to facility records. Data were analyzed using an inductive, open-coding approach to thematic analysis. Ten of the respondents (7 out of care, 3 in-care) had stopped and re-started treatment before the interview date. One of the most important factors influencing adherence and retention was the strength of women’s support systems. In contrast to women in-care, most out-of-care women lacked emotional and financial support from male partners, received minimal counseling from providers at initiation, lacked designated guardians to assist with medication refills or clinic appointments, and were highly mobile. Mobility led to difficulties in accessing treatment in new settings. The most common reasons women re-started treatment following interruptions were due to providers’ counseling and encouragement and the mother’s desire to be healthy. Improved counseling at initiation, active follow-up counseling, women’s economic empowerment interventions, promotion of peer counseling schemes and meaningful engagement of male partners can help in addressing the identified barriers and promoting sustained retention of Option B+ women.

## Introduction

In 2011, Malawi introduced the Option B+ strategy for prevention of mother-to-child transmission (PMTCT), becoming the first country to offer lifelong antiretroviral therapy (ART) for all HIV-infected pregnant and lactating women regardless of their CD4 cell count or clinical stage. In the first year of implementation of this strategy, there was a 7-fold increase in ART initiation of pregnant and lactating women [1]. From 2009 to 2016, the proportion of HIV-positive pregnant women receiving ART increased from 21% up to 87% [2–3].

However, recent findings have highlighted the challenges of Option B+ in regards to early loss to follow-up (LTFU). For example, a study that analyzed nationwide, facility-level data in Malawi found a LTFU rate of nearly 24% (95% CI 22.6–25.3%) in the first 6 months in high patient volume facilities, with most losses occurring in the first 3 months [4]. An observational multi-facility cohort study in Malawi had comparable findings of high LTFU (22%) in the first year of implementing Option B+ [5] while a study from one facility in Malawi further clarified that 47% of LTFU women only collected their drugs at the time of initiation but never returned to the same clinic to refill their drugs [6]. Published works from other sub-Saharan African countries also show similar gaps regarding retention in care [7–8]. Some of the factors associated with LTFU under Option B+ included younger age at initiation, [5–6,9] being pregnant (compared to lactating) [6], starting ART on the same day of diagnosis [4,7,9], lack of disclosure [7], missing CD4 cell counts at ART initiation [9], having less than a secondary education [7], and receiving care at high volume facilities [4–5, 9].

In terms of adherence patterns, a multi-facility cohort study in Malawi that defined adequate adherence as ≥90% determined close to one-third of women enrolled in the Option B+ program inadequately adhered to ART during pregnancy and lactation, and that those who initiated under Option B+ were 1.5 times more likely to inadequately adhere when compared to women who were not pregnant and not lactating [10]. The study cited that risk factors for inadequate adherence included starting ART under Option B+, at a younger age, or at a government-managed district hospital or health center (when compared to facilities managed by faith-based institutions) [6,10]. A review of 40 PMTCT studies from sub-Saharan Africa summarized poor HIV/ART/PMTCT knowledge, low maternal education level, psychological challenges following diagnosis, stigma, and fear of disclosure as common barriers to adherence and retention [11]. Degree of partner and community support was another key factor affecting ART uptake for PMTCT, as were facility-related factors, such as weak patient-provider interactions, service accessibility and staff shortages.

In the context of Option B+, there is limited published evidence from qualitative studies that explore barriers to retention and adherence from patients’ perspectives. Barriers identified in the few existing published qualitative studies include inadequate counseling [8,12], ART-related side effects [13–14], fear of disclosure [15–16], HIV-related stigma [15–16], skepticism about lifelong treatment [12] and poor patient-provider interactions [15–16]. Among these studies, few considered perspectives directly from Option B+ women who had already defaulted from HIV care [13–15]. Yet, women who discontinue their HIV care offer a unique and valuable perspective. This study sought to acquire a deeper understanding of factors affecting women’s treatment-related decision-making during periods of pregnancy and lactation. The insights gained from this study are expected to inform future interventions that aim to increase uptake and retention of pregnant and lactating women initiated on ART, while decreasing the number of new infections in infants born to HIV-infected women.

## Methods

### Study setting

This study was conducted at Bwaila District Hospital in Lilongwe, Malawi. Bwaila District Hospital has the busiest maternity unit in Malawi, with more than 50 pregnant women admitted for labor and delivery each day in 2015. The antenatal clinic (ANC) at Bwaila Family Health Unit (FHU) has been initiating HIV-positive pregnant and lactating women on treatment since September 2011. Similar to other high volume facilities in Malawi, routine ART services delivered to HIV positive women at Bwaila FHU are captured in a real-time touch screen electronic medical records (EMR) system. As part of the routine practice, informed consent for tracing in the event of missed appointments is obtained from patients at their initial clinic registration. Follow-up status of all current patients receiving ART is one of the program quality indicators that is reviewed on a monthly basis. Patients who have missed their appointment by more than 21 days are referred to the in-house patient tracing project called Back-To-Care (B2C), managed by Lighthouse Trust. The B2C staff employ text messages, phone calls and field visits to reach patients, remind them about their missed appointment and arrange for their return to care before they meet the national definition of ‘defaulter’ which indicates missing a scheduled appointment by more than 2 months [17].

### Study population and sampling

Participants in this qualitative study were pregnant and lactating women initiated on ART at Bwaila FHU, under Option B+ strategy. They included women who were out of care (defined as those who initiated ART, but missed their scheduled appointments by more than 21 days without notifying hospital staff); and also women who had been retained in care. These two categories were used to examine any potential differences in factors affecting women’s treatment-related decision-making. Recruitment of respondents who were out of care was done during routine active tracing conducted by the B2C team through phone calls or home visits. Among women in care, pregnant women who enrolled and remained in Option B+ care for more than 3 months and lactating women who enrolled and remained in Option B+ care for more than 6 months were eligible to be interviewed. Duration of care for pregnant women was shorter due to the short pregnancy period (9 months) compared to the longer lactation period that is customary in Malawi. Respondents were recruited until information redundancy was reached [18]. Recruitment of women in care was done at the end of their routine ART pick-up visit at the ANC of Bwaila Hospital by health care providers (HCP) who were mostly nurses. Women who did not consent for active follow up at their initial clinic visit, and those that refused further communication from the clinic after being contacted by the B2C staff regarding their missed appointment were not interviewed.

### Data collection and management

Between July and October 2015, 39 in-depth interviews were conducted using semi-structured interviews to understand women’s lived experiences and expressed perspectives with ART initiation, adherence and retention in care at Bwaila FHU. In-care women were recruited by HCPs after their routine ARV refill visits. The interviews were conducted by an independent evaluator in a private room at Bwaila Hospital. Women who were out of care were recruited by the B2C team, and interviewed by an independent evaluator at a location identified by the respondents. These included respondents’ homes, a location in their community, and a private room in Bwaila Hospital.

All participants were asked about their experiences of HIV testing, counseling, ART initiation, follow-up care and types of support needed to remain in care. Probing questions were used to articulate when and why specific events occurred (while pregnant, at birth, during lactation). Women who were out-of-care at the time of the interview were also asked about their reasons for discontinuing their HIV care, and reasons for re-starting or intent to re-start ART. Interviews were conducted in Chichewa (local language in Malawi) by three qualitative data collectors, audio-recorded, transcribed and translated into English. Data quality was ensured through training of data collectors, piloting and refining of data collection tools and ongoing review of transcripts by a senior researcher during data collection.

### Data analysis

The analysis intended to understand barriers and facilitators of adherence to ART and retention in care. Two researchers independently reviewed the transcripts several times, identified patterns and developed an initial codebook including clear definitions for each code, and examples of their application. The codebook was then independently applied to a smaller set of transcripts and reviewed by a third researcher. Coding disagreements were resolved through discussions and used to refine the codebook. This process was conducted two more times, first with a different selection of transcripts and then, after finalizing the codebook, with the full set of transcripts. Based on the coded data and guided by the study’s research question, themes were generated, discussed and agreed upon by the research team.

Data were analyzed using an inductive, open-coding approach to thematic analysis [19]. Atlas.ti v.7.5.12 (Scientific Software Development GmbH, Berlin, Germany) was used to support coding, analysis and data organization. Descriptive data matrices were also developed to summarize key respondents’ information and facilitate the process of comparison across the various respondent categories. Descriptive statistics were used to summarize respondents’ sociodemographic data.

### Ethical considerations

All research team members were trained on the importance of maintaining confidentiality and protecting the privacy of respondents. If potential respondents decided not to participate, they were informed that their decision would in no way affect the quality or type of care they would receive at Bwaila Hospital or other health clinics. This was communicated with respondents during the informed consent process, in addition to offering them an English and Chichewa version of the consent information sheet. Prior to commencing interviews, the interviewer briefed each respondent about the study and obtained verbal informed consent to conduct and record interviews. Signed consent was not obtained to avoid records that could link respondents to this study. A yes/no checkbox was used by interviewers to indicate consent of participants. Interviewers did not document names or any other personal identifiers in their notes. A study-specific ethical review and approval was obtained from the National Health Sciences Research Committee in Lilongwe, Malawi.

## Results

### Study population

Of the 39 women interviewed, facility records showed that 14 were in care while 25 were out of care from Bwaila FHU at the time of recruitment. The 14 participants who were in care included 6 pregnant and 8 lactating women, while the 25 women who were out of care included 12 pregnant and 13 lactating women. By the time the interviews took place, 2 women who were out of care and categorized as pregnant in facility records had given birth and were lactating. The majority of the women (n = 36) initiated ART due to pregnancy. Additional sociodemographic characteristics, such as participants’ marital status, time of HIV diagnosis, reason for ART initiation, time of initiation after of diagnosis, status at time of stopping ART are summarized in Table 1. Two individuals reported not having disclosed their status to anyone, while 4 of the 34 women that had partners indicated not disclosing to their partner.

**Table 1:** Characteristics of interview participants (N = 39).

Characteristics	N	%
**Location of interview**		
At Bwaila Family Health Unit (FHU)	31	79
In the Field	8	21
**Age**		
<25 years old	18	46
≥25 years	20	51
Unknown	1	3
**Marital status**		
Married/in a relationship	34	87
Single/divorced	4	10
Widowed	1	3
**Time of HIV Diagnosis**		
During pregnancy or lactation	32	82
Before pregnancy or lactation	7	18
**Status at time of ART initiation**		
Pregnant	36	92
At birth	1	3
Lactating	2	5
**Time of Initiation**		
Same-day initiation	31	79
Delayed initiation	8	21
Status at time of stopping ART (n = 28*)		
Stopped while pregnant	18	64
Stopped while lactating	10	36
**Status at time of interview**		
Pregnant	16	41
Lactating	23	59
**Disclosure to others**		
Disclosed to someone	37	95
Disclosed to no one	2	5
**Partner disclosure**		
Disclosed to partner	30	77
Did not disclose to partner	4	10
No partner	5	13

*Includes all LTFU women and 3 women in care who interrupted treatment for some period of time.

### Awareness of Option B+ and perceived benefits

While none of the respondents used or immediately recognized the term “Option B+”, both in-care and out-of-care women were familiar with certain aspects of the strategy. For example, all respondents reported that HIV-infected pregnant and lactating women should initiate ART as soon as they are diagnosed with HIV. Most patients noted the importance of taking their drugs at the same time daily; however, less than a quarter of respondents (1 pregnant and 3 lactating women who were in-care, and 3 lactating women out-of-care) mentioned the need for lifelong commitment to taking antiretroviral (ARV) drugs.

“*It just happened that I stayed very far away from the hospital at my village. Had it been that it [the hospital] was nearer, I could have walked myself there to access the drugs. […] I do understand the benefits of PMTCT. You might think that I did not understand just because I stopped taking the drugs. I understand the all recommendations and I know that you don’t have to skip days. I know you have to be taking them for the rest of your life*.”

(R27, 21 years old, lactating, out of care)

All women described acquiring HIV, ART, and PMTCT-related information from health facilities, and most saw health facility and HCPs as the most trusted source of information on treatment. Less commonly mentioned sources of information included radio, television, discussions with family and friends, organized support groups and schools.

When women spoke about the counseling they received from providers at initiation, they most often described being told of the importance of starting treatment to protect the baby. Some also said they were informed about ART’s advantages for their own health. None mentioned providers discussing ART in the context of protecting one’s partner (i.e., treatment as prevention). Some respondents commented that being on treatment reduced stigma towards the mother and baby, as a healthy appearance allowed them to hide their HIV status. Only a few women mentioned the benefit of being able to safely breastfeed longer.

### Factors affecting women’s decision-making during initiation

#### Same-day initiation

Most women (n = 31) initiated treatment on the same day, saying they “accepted” to start the drugs, initially motivated by providers who emphasized the need to protect the baby’s health. However, data suggest these decisions were rarely straightforward or influenced by one single factor; rather, the process was fluid and levels of acceptance shifted over time. Initiation of treatment seldom signified women’s acceptance of their positive status and their need for ART. Some women suggested they did not consciously “choose” to begin treatment. This was especially common among those who were asymptomatic or had previously tested negative.

“*I*: *When you were told at first to start taking the drugs, how did you handle it?*

*R*: *I didn’t want to, but I had no choice. They told me it was for the sake of my baby and I accepted*.

*I*: *How did you feel when you came for antenatal care and then you were told to start taking the drugs?*

*R*: *I did not accept it, because all this time I had been fine*.”

(R25, 22 years old, pregnant, in care)

Nearly all respondents described initiating treatment with trepidation and resignation, as they coped with the psychological repercussions of their diagnosis. They expressed being overwhelmed, in denial, worried about their partner’s reaction, afraid of potential physical and psychological side effects of ART and/or fatalistic about the diagnosis.

“R. I felt pain in my heart because of what I was told…I asked myself whether I should hang myself.

I. What made you accept the situation? What motivated you?

R. I just said to myself that I am not the first one to experience this problem.”

(R31, 17 years old, lactating, out of care)

“I: When you were told, how did you handle it?

R: I did not believe it…

I: Why? What made you not believe the results?

R: I didn’t believe that it was true because of the way I was leading my life.”

(R18, 25 years old, pregnant, out of care)

Both out-of-care and in-care women reported acceptance was facilitated by realizing that they were “not the first” person to battle HIV and they could still live a long productive life on ART.

“I just left [the hospital] and put the drugs in my bag and went home. When I arrived [home] I slept because my head was not working properly as to how this [diagnosis] could be. Yet, I accepted it, and then I called a friend and explained to her that my head is not working, the disease, the problems I am passing through… [She] told me, ‘Don’t worry. You are not the first one. Here, there are many people who are taking treatment. Accept it. You will see that you are living for years without dying, and also not appearing as you have this disease.’ That is how she encouraged me. It took a long time to accept but still I was taking the treatment.”

(R7, 27 years old, lactating, in care)

Similarly, some in-care and out-of-care women were motivated to initiate treatment due to personally knowing or seeing other people living with HIV in the community who were on ART and looked healthier as a result, or who were not on treatment and looked unwell or had died.

“[Where I used to live], I have seen a lot of other people with the virus and on HIV medication. They were living a better life. So I thought that I should also start taking [ARVs] and I will be okay. Some of them were my relatives and also other people. I saw that it was better to take the drugs than not take them. There were some other people who were not taking the drugs. They were just keeping them and now they are dead.”

(R35, 23 years old, pregnant, out of care)

While it was rare that women described immediately accepting the need to start ARVs without some amount of hesitation, the few in our study who knew of their status before becoming pregnant found acceptance of treatment easier. One woman who knew about her HIV infection before her ANC visit mentioned, “I accepted without any problem, because l already knew my status. It was not hard for me” (R37, 23 years old, pregnant, out of care).

#### Delayed initiation

Delayed initiation of ART was noted by 8 respondents. The length of reported delay was an average of 10.6 days [range 1–30 days]. Most women described psychological barriers as the cause of delay, including denial of their diagnosis and fear of taking drugs. Common among this group of women was that they all refrained from disclosing to anyone for a long period of time following their diagnosis and/or they lacked strong support networks.

Facility-level factors were described by 3 other women as causes of delayed initiation. One woman reported being diagnosed during her 3^rd^ month of pregnancy, but the provider told her to return 2 months later because her pregnancy was “too young” to start treatment. Another explained that after she was diagnosed, she was told to come back after a week to take part in a related research project and to pick-up her ARVs then. The other respondent learned she was diagnosed at a facility that did not provide antenatal care but provided her Bactrim for a week with a transfer slip to Bwaila ANC. The average duration of such delays was 24.7 days [range 7–60 days].

### Facilitators of retention in care

Of the 14 respondents who were in care at the time of interview, most (n = 11) reported no interruptions to their treatment. One of the primary facilitators for retention was the existence of a strong support system, including designated “guardians” or treatment supporters who offered treatment-related encouragement and assisted with medication pickups. Most had disclosed to more than one person, in order to ensure help would be available when needed.

For women who were in relationships, an important aspect of their support system was having a supportive husband or partner. Partners aided women’s retention and adherence by motivating them to start swallowing the prescribed ARVs, reminding them to take their drugs daily, allaying treatment-related fears, and assisting them financially to remain in care (including provision of transport money).

“I: Ok, did you select a guardian to help you manage the drugs?

R: Mmm [agreeing]…My husband.

I: What does your husband think about you taking the drugs?

R: He is the one who reminds me to take the drugs…”

(R26, 30 years old, pregnant, in care)

A few of the women who were in care, but did not have partners, described receiving strong emotional and financial support from their families, especially from their mothers.

HCPs were another important source of support for women in care. All respondents described being motivated by HCP counseling and encouragement, and spoke favorably about their encounters with them. Women reported that providers helped them to understand their condition, realize the importance of ART adherence and develop strategies to maintain adherence. Furthermore, respondents who remained in care often expressed a positive, proactive attitude about learning their status and being on ART. Several women who remained in care commented how being HIV-infected “is not the end of everything,” and understood they could live a long life on ART. They also explained it was better to know their diagnosis because it enabled them to seek help and address the disease, as opposed to remaining unaware and suffering potential health consequences.

“I consider [being on treatment] to be a very important thing because by now my child could have been infected. I think taking the drugs is a wise decision. There are a lot of people who think their bodies are fine and don’t know their status. But if someone knows their status, they know what to do and can go on in life.”

(R1, 21 years old, lactating, in care)

Social networks also played a major role in promoting in-care women’s gradual psychological shift from resignation to a deeper acceptance of their status. This consequently facilitated retention and adherence, as women adopted new HIV-positive identities; identities inextricably linked with ART: “I have not seen anything that can stop me from taking the drugs, because my life is now in the drugs” (R21, 19 years old, pregnant, in care).

Though almost all women who remained in care experienced adverse side effects from ART, they tended to emphasize the benefits of treatment over its drawbacks. Several respondents described being motivated to remain on treatment due to health improvements, such as having an HIV-negative baby and/or feeling sick prior to taking drugs and better after starting them.

Some women also demonstrated an awareness of the consequences of stopping ART, fear of which was a motivating factor to remain on treatment. As one woman noted, “If you stop or give up [taking ARVs], it means the diseases will not leave you. Continue taking the drugs so that the virus can be defeated” (R5, 19 years old, lactating, in care). Furthermore, none of the in-care women described any sort of transportation barriers or clinic access issues, nor did any of them cite travel or attempts to self-transfer to another facility, which otherwise could have impacted their ability to be retained in care.

There were 3 women who remained in care but reported interruptions to their treatment. Reasons for interruptions included side effects and forgetting to take drugs. All 3 sought hospital support and reported that providers successfully helped them manage their challenges, thereby reducing their risk of LTFU. As one woman who interrupted treatment due to the side effects of nightmares explained, “If I had not come here at the hospital to ask [about side effects], I would have stopped [ART]. The animals [in the nightmares] were very scary” (R8, 29 years old, lactating, in care).

### Reasons for discontinued HIV care

In stark contrast to women in care, nearly all out-of-care respondents (22/25) described lacking strong support systems, such as partner and family support and/or designated guardians, to help them cope with their diagnosis and assist with treatment. Lack of partner support was mentioned by more than half of out-of-care women. All but two disclosed to their partners, though few were supported in response. Women frequently reported conflicts with partners, including those who refused to accept the diagnosis, those who placed blame on the wife/partner, and/or were physically or verbally abusive. One woman’s husband hid her health passport after she revealed her status, and another forced his wife to go to her family’s village.

“When I went home after being told that I have the virus, the situation was bad. He said, ‘You went to the hospital and have been found HIV positive. How has that happened?’ There was a quarrel and he said I have to go [to the village] and I told him, ‘I have been tested and know my status. You haven’t tested and don’t know your status. Yet, you are sending me away as if am the one who has made it that I should have the disease.’ He said ‘No, you were not behaving well.’ There was a misunderstanding.”

(R16, 28 years old, pregnant, out of care)

A number of women in relationships expressed a strong desire for their partners to get tested, even asking them many times, though their partners refused to do so. Some discovered their partners had been tested, but never disclosed the results. Respondents expressed feeling isolated and alone as a result of these situations, particularly when they suspected their partners were also HIV-positive. Other noted barriers were partners who did not provide emotional support or encouragement around ART, those who lived and worked far away, and one partner who refused to assist with or financially support the pregnancy and baby. For many women, the challenge of minimal or no partner support was exacerbated by the absence of relatives living nearby, most of whom resided in the woman’s home village. Without supportive partners and/or nearby relatives, designating a guardian was difficult. More than a third of out-of-care women said they did not have an assigned guardian to assist them with appointments and medication refills.

“I: Why did you decide to disclose to your husband only?

R: If I had a relative [here] I could have disclosed to him or her, but I don’t have any.

I: You did not choose anyone or a guardian to help you manage the drugs.

R: Hmmm mmm [no]…A relative would have come to collect the drugs for me, but a stranger cannot agree to do that.”

(R19, 35 years old, lactating, out of care)

Mobility was another frequently reported barrier to women’s retention, with more than one-third of out-of-care respondents citing it as the reason for interrupted care and treatment. Lack of partner support, particularly given the context of gender inequality, often meant that women were vulnerable to displacement following conflict with male partners. Tending to family obligations in women’s home villages, such as the need to care for a sick relative, was also a common cause of movement. Women faced difficulties accessing ART at their destination due to being in a rural or new setting, lacking a health passport, and facility-related work standards that were not consistent with the national guidelines, including refusal of treatment without a transfer letter from the facility where treatment was initiated. Two-thirds of the women who stopped treatment due to mobility did so postpartum. All the women who stopped ART during lactation cited mobility as the primary cause of discontinued care. Almost all of lactating out-of-care women described having minimal or no social support from their male partners.

The majority of respondents described being dependent on their male partners for financial support. Consequently, access to treatment was interrupted or postponed when their partners lacked money for transportation or did not view it as a priority.

“Sometimes I want to come to the hospital, but due to lack of transport money I don’t. If money is available it may be easy for me to take the drugs […] there are no relatives around [to provide transportation assistance], as they are all in the village and I stay with my husband. So if he finds money, I can come to collect the drugs.”

(R18, 25 years old, pregnant, out of care)

Individual-level barriers to remaining on treatment were also cited, though much less frequently. In these cases, 5 respondents described an intention to stop their treatment motivated by side effects such as dizziness, rapid heartbeat, nausea, vomiting, hallucinations and/or skin sores. Unlike those who remained in care, none of the out-of-care respondents who ceased treatment due to side effects told providers about them or sought hospital assistance to manage them. Some even described avoiding treatment follow ups as a result. As one respondent stated, “I hated the [ARVs]. Whenever I took the drugs, I felt dizzy and felt sick. So, I thought that my body has not accepting that I should be taking the drugs” (R28, 20 years old, lactating, out of care). Such attitudes were compounded by negative perceptions about treatment providers among the out-of-care women. Multiple women said they would not return to the facility out of fear that treatment providers would “shout at” or “insult” them after they had missed their last appointments.

### Reasons for re-starting interrupted treatment

**Fig 1:**
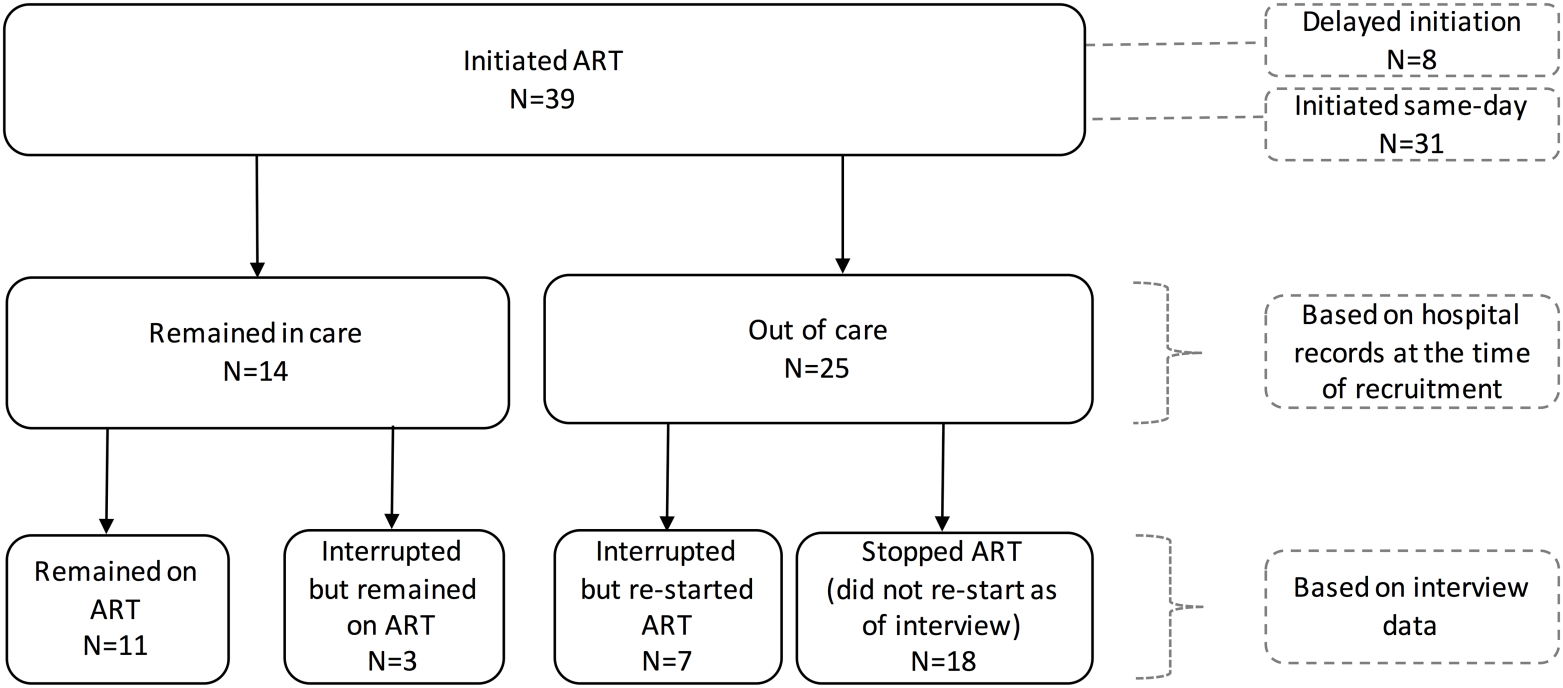
Study sample of pregnant and lactating women who initiated Option B+ services at Bwaila Family Health Unit (BFHU), Lilongwe, Malawi.

Despite the treatment status of participants documented in hospital records at the time of interview, 3 in-care and 7 out-of-care women had stopped and restarted treatment in the past (Fig 1). The 10 study participants who reported stopping and re-starting ART mentioned HCP counseling and encouragement and the mother’s desire to be healthy as their main reasons for re-starting an interrupted treatment. Some respondents described how trust in HCPs enabled them to disclose treatment-related fears and seek advice on the challenges of taking ARVs. Other common reasons for re-starting treatment were fear of or actual decline in health while not on treatment, and re-gaining access to ART, either due to securing transportation money or after returning from travel to locations where they could not re-fill their ARVs.

Two women who re-started ART did not perceive that they had actually “stopped” treatment, because they returned to care or re-started treatment on their own, after brief unintended interruptions. Three of these women were mislabeled as out-of-care as they reported self-transferring to another facility and were back on treatment at the time of the interview, though this information was not yet captured as such in hospital records. The duration of treatment interruptions for in-care women was, on average, 4 days [range 2–7 days], while interruptions for women categorized as out-of-care lasted for 1.5 months [median duration 45 days, IQR 10–81].

When asked about the type of support they needed to achieve better adherence and retention, out-of-care women requested additional counseling, specifically on taking ARVs, advice on how to remember to take prescribed drugs daily, information on side effects, and assistance to remember their appointment dates. Some women also described needing help from HCPs on convincing their partners to get tested: “There is a need to provide assistance as to what should be done when a woman goes to the hospital and tests positive, yet the man did not go” (R16, 28 years old, pregnant, out of care). Several respondents also remarked that they needed transportation assistance to reach the clinic, and a number of women stated they wanted help from others to collect their drugs.

#### Women who stopped ART and never re-started

Most out-of-care women who did not re-start treatment at any point described facing a combination of major barriers that prevented ART adherence at the time of their interview. This included lack of support system (no guardian, living away from family members) and ongoing transportation barriers. Only one woman in the study openly expressed a lack of intention to re-start treatment citing religious reasons: “By faith, it is possible to remove any problem in your body” (R14, 35 years old, lactating, out of care). She was also one of only 2 respondents who did not disclose to anyone. Another woman reported testing negative while visiting another facility and never re-started afterwards.

## Discussion

Currently, there are 21 Global Plan priority countries across sub-Saharan Africa in the process of scaling-up Option B+ programs [20]. Given the strategy’s rapid roll out in the region and the challenges of inadequate adherence and early LTFU highlighted in recent quantitative studies [4–5,7–8,10], there is an urgent need to enhance understanding on how to improve ART adherence and retention. Qualitative research is useful for exploring meanings behind quantitative findings [18]. In this case, investigating individual perspectives on treatment-related decision-making and the context in which those decisions were made yielded a greater awareness of why some women remained in care and on treatment, while others did not. This qualitative study explored the barriers and facilitators for treatment adherence and retention faced by pregnant and lactating women initiating ART for PMTCT. The examination of stopping and restarting treatment from both in-care and out-of-care respondents offers a unique and valuable perspective towards addressing sustained treatment after women are initiated on ART due to pregnancy or lactation.

### The role of support systems

Our study found the strength of women’s support systems to be one of the most important factors in influencing ART adherence and retention. Social networks comprised of male partners, family, friends and HCPs helped women to cope with the psychological consequences of HIV diagnosis, accept their status and encouraged adherence through emotional and financial support. Several other studies from the region have demonstrated the effect of social support on uptake and ART adherence. In PMTCT programs, male partner involvement has been shown to statistically increase women’s likelihood to accept HIV testing, initiate treatment, remain in care, and decrease the risk of infant death due to vertical transmission [21–25]. Various studies have identified social support as a key factor in achieving high ART adherence. One study found patients drew upon their “social capital” in order to remain adherent [26], while others showed how ART adherence is facilitated by strong social networks [27–29] and positive patient-provider relationships [30–33], especially for patients with low socioeconomic status [34].

### The role of counseling

Our findings suggest the quality of HCP counseling was inconsistent and inadequate for many women initiating ART under Option B+. Counseling can play a crucial role during the diagnosis phase and throughout the continuum of care; however, to be effective, it must address the psychological, emotional and informational needs of the patients. The majority of women who were out of care described receiving minimal counseling at initiation, with content that focused on the need to start ART and its importance for protecting the baby. Very few out-of-care women described receiving any amount of psychological support from providers around making the significant decision of starting lifelong treatment. On the other hand, several in-care women spoke positively about the advice and encouragement they received at initiation, suggesting the benefit of enhanced counseling at the diagnosis phase. Similar information had emerged from focus group discussions with HIV-positive women in Malawi [35]. Many turned to other women living with HIV or female relatives to seek necessary support regarding initiation and follow up of treatment. As demonstrated in our study, the decision-making process of initiating ART under Option B+ was psychologically and emotionally complex, especially for women who were unable to seek adequate support elsewhere. This stage has been described as a “triple burden”: transitioning into pregnancy, accepting their HIV diagnosis, and acknowledging the immediate need to begin lifelong ART, often while asymptomatic [36]. In our study, some women expressed an additional burden of coping with the notion that their partners were unfaithful.

A number of studies have demonstrated the importance of HIV counseling in significantly improving patient uptake and retention [37] and viral suppression [38]. Qualitative studies have also found that provider support and counseling facilitated patients’ retention in care [29] and specifically PMTCT care [39,40]. Gourlay et al found that effective provider communication, knowledge transfer and provision of psychosocial support promoted women’s uptake of PMTCT services [40]. It was further suggested that psychosocial counseling acted as a catalyst to enhance family support, both of which contributed to greater likelihood of patient retention in PMTCT programs.

### Recommendations

Our findings and recommendations on Option B+ lend support to and expand upon a recently published theoretical framework which considers retention in HIV care from the patient perspective. The framework posits that sustained retention is contingent upon 3 related processes: 1) patient activation, 2) social normalization and 3) livelihood routinization [41]. The process of patient activation involves a psychological shift from “passive recipients of care” to patients who accept their status, become more knowledgeable about their condition and engage with HCPs to co-manage their care. Based on our findings and related evidence from other studies, enhanced counseling is essential to this process.

Many women in this study failed to describe the benefits of Option B+ aside from the baby’s and mother’s health, and few mentioned the need for lifelong treatment. Ensuring that counseling at initiation covers education what HIV infection is, the importance of ART, the need of long-term adherence for the lifelong health of the mother, the reduced transmission risk for future pregnancies, and treatment as prevention in serodiscordant relationships may give women the additional information needed to encourage them to start and remain in care [31]. In these sessions, there may also be a need to articulate the common side effects that should be anticipated, how to identify them if they occur, and the collaborative role of HCPs in managing them, while allowing women to speak openly about challenges and clarify information [42].

In low socioeconomic areas of sub-Saharan Africa, our study has noted that social capital is critical for women initiating treatment on Option B+, many of whom are young, financially dependent on male partners and/or family, and faced with the unique circumstances of managing a “triple burden” [36]. Assistance with disclosure processes, encouragement of male partner participation, coaching of women in communicating their status to others and describing their need for support to family and friends may promote social normalization after initiation on lifelong treatment [34]. Disclosure to male partners can be exceptionally difficult due to feared repercussions such as displacement, abuse, divorce and financial vulnerability [43]. Other studies have also stressed the importance of creating a conducive environment for male partners when attempting to increase their participation, as men often feel uncomfortable attending ANCs, which are seen as women-only spaces [44].

Upon initiation of ART, linking women with peer or mentor mothers who are living with HIV and emphasizing the need to designate a guardian may facilitate the establishment of new social networks for support [45]. Several respondents in this study were motivated by personally knowing others with HIV and on ART. This notion is supported by a PMTCT-focused literature review which summarized the success of several peer counseling approaches, including the use of “mentor mothers,” which led to improved coping strategies, increased clinic attendance and lactation duration, and reduced depression [23].

Roy et al suggest that sustained retention relies on patients’ ability to successfully integrate care and treatment activities with livelihood demands [41]. While this aspect of the framework is valuable, we also believe it is necessary to re-examine it through a gender lens. Most women in our study relied on others for financial support (usually male partners). Couples lacked interdependence, which indicates women may experience added barriers to sustained retention and limited control over livelihood routinization due to violence or dissolution of relationships [46]. Aside from financial dependencies, many women turned to other relatives to seek necessary support regarding initiation and follow up treatment. Disruptions in relationships within their support systems, or being expected to provide care for family members that lived far away, routinely resulted in mobility of women away from their residence, which in turn affected women’s retention in care [6]. Ensuring providers discuss possible mobility at initiation and during follow-up, and offering options to collect extra drugs prior to travel, may mitigate the risk of LTFU in societal structures where women are expected to care for family members who live away from where they live and for women who may not have supportive partners.

### Study strengths and limitations

This was a small, qualitative study at one high-volume facility in the city of Lilongwe, and therefore not generalizable to the broader population. There were, however, some parallels with findings from other studies with similar populations in urban centers in Malawi [12–13,15]. Most women were interviewed at Bwaila Hospital where they initiated care, which may have resulted in biased feedback if they felt less open to share negative feedback about providers. Interviewers tried to minimize such bias through the informed consent process, explaining that participants should feel free to share their honest opinions and that participants’ comments would not in any way affect their quality of treatment. The exclusion of women who did not consent for active follow up at initial clinic visit or refused further communication may have resulted in a selection bias that resulted in omission of perspectives from women who do not welcome support from health facilities. In order to avoid social acceptability bias, out-of-care women were deliberately interviewed before they were provided enhanced counseling which is routinely conducted for individuals who restart an interrupted treatment. After interviews were conducted, out-of-care women received adherence counseling that also led them to restart their care and pick up ARVs per HCP guidance.

Our study focused on pregnant and lactating women on ART and highlighted facilitators and barriers to sustained ART. It is important to note that both in-care and LTFU women reported having interrupted treatment in the past, which is an area that is not often captured in cross-sectional quantitative retention reports. A mixed methods study can elaborate on the magnitude of and reasons for treatment interruption in women who initiated lifelong HIV treatment due to pregnancy or lactation.

## Conclusion

In order to address the barriers and promote optimal ART retention, strategies that recommend universal initiation of ART in asymptomatic individuals need to also promote improved counseling at initiation along with a case-based management plan that considers the individuals’ plans for disclosure and social support needs. Initial and active follow-up counseling regarding side effects, women’s empowerment interventions, promotion of counseling schemes that use mentor or peer mothers, and meaningful engagement of male partners may promote sustained ART retention among Option B+ women.

## Supporting information

S1 File Interview guide. (DOCX)
