# Supplementary material for: Exploring factors associated with ART adherence and retention in care under Option B+ strategy in Malawi: A qualitative study
Source: PLoS One. 2017 Jun 21;12(6):e0179838. doi: 10.1371/journal.pone.0179838 (PMC5479573; doi:10.1371/journal.pone.0179838)
Supplement: S1 File — (DOCX) [file pone.0179838.s001.docx]

# Interview Guide

## Knowledge assessment

1. What can be done to prevent transmission of HIV from a mother to her child if the mother is known to have HIV in her blood?
   1. Where did you learn about this? *(Probe: If they have received information on PMTCT from single or multiple sources. Where did they receive the most information about? Which information sources do they trust the most?)*
2. Do you think there are benefits of taking HIV medications for an HIV positive pregnant or lactating woman?
   1. If yes, what are the benefits specifically to the mother?
   2. If yes, what are the benefits specifically for the infant?
   3. Are there any disadvantages? If yes, what are they? *(Ask this question even if they mention benefits)*
3. When should an HIV positive woman take HIV drugs?
   1. What are the benefits of taking HIV drugs during pregnancy?

*(Probe: what are the challenges of doing so?)*

- 1. What are the benefits of taking HIV drugs at birth?

*(Probe: what are the challenges of doing so?)*

- 1. What are the benefits of taking HIV drugs after birth?

*(Probe: what are the challenges of doing so?)*

- 1. What are the disadvantages of taking HIV drugs at any of these times (before, at or after birth)?

1. Have you heard of Option B+?
   1. If yes, what is it and where did you hear about it?

## Self-assessment

1. When did you find out about your current HIV status? *(Probe: Did you learn before your first antenatal visit for your most recent child? Did you learn at your first antenatal visit for your most recent child or did you learn after giving birth?)*
2. When were you first told about the need to take HIV drugs? (*Probe: Did you receive ARVs at the antenatal clinic during your first antenatal visit? Was this recommendation done before that visit? Was a recommendation to start treatment done on the same day that you learned your HIV positive status? How did you handle the news?*)
3. What do you think about being given HIV drugs in your case? *(Probe: What are the benefits? What are the disadvantages? What did you have to weigh and balance?)*

## Social support

1. Who are the closest people that you rely on for social support? *(Probe: Do you work/ have a business, or do you rely on your husband, family members or friends?)*
2. Which of your support systems would you rely on during your pregnancy, birth and after birth?
3. From your support system, who do you trust enough to disclose your HIV status to them? *(Probe: Have you selected a guardian to help you manage ART? How would your taking HIV drugs affect the social support you get/anticipate to get during your pregnancy, birth and after birth?)*

## Intent to remain in HIV care

1. When you received the HIV drugs prescribed to you at the clinic, what did you do first?
   1. [if start taking them] How soon did you start taking them after your clinic visit? What was your motivation in taking (swallowing) them? *(Probe: Personal drive, focus on health, trust in health care providers, support from family and friends)*
   2. [if other plans] What is your reason for this plan? *(Probe: Lack of personal drive, fear of drug side effects, lack of understanding, distrust in health care providers, other issues with the clinic, fear of disclosure, lack of privacy, lack of understanding from family and friends)*

***[The following questions are for women who have stopped/never started their ARVs]***

1. Have you ever taken (swallowed) the HIV drugs prescribed to you?
2. If yes, when exactly did you begin taking (swallowing) your ARVs?
3. If stopped or taken intermittently, when exactly did you stop taking (swallowing) your ARVs? *(Probe: After how many months, after how many clinic visits, while pregnant or after giving birth?)*
4. If stopped or taken intermittently, what caused you to stop/interrupt taking (swallowing) your ARVs? *(Probe: Did your situation at home change in some way, social or financial, was it an issue with the clinic or was it an individual choice?)*
5. What changes would make you consider re-start taking your HIV medications? (Probe: Changes at home, More privacy, more money, switching clinics, feeling more comfortable at the clinic)
6. What kind of support can help you make an informed decision? (Probe: a counselor to come home to talk to your husband/family members, peer support,)
7. What can the staff at the clinic do to make it easier for you to understand the process and benefits of PMTCT?
8. What kind of support would make it easier for you to take (swallow) ARVs?
